# Identification and characterization of a novel formaldehyde dehydrogenase in *Bacillus subtilis*

**DOI:** 10.1128/aem.02181-23

**Published:** 2024-10-29

**Authors:** Vivien Jessica Klein, Susanne Hansen Troøyen, Luciana Fernandes Brito, Gaston Courtade, Trygve Brautaset, Marta Irla

**Affiliations:** 1 Department of Biotechnology and Food Science, NTNU Norwegian University of Science and Technology, Trondheim, Norway; Washington University in St. Louis, St. Louis, Missouri, USA

**Keywords:** *Bacillus subtilis*, formaldehyde detoxification, formaldehyde oxidation, formaldehyde dehydrogenase, protein expression, protein purification

## Abstract

Formaldehyde is a known toxic compound, and functional formaldehyde detoxification is crucial for the survival of all living cells. Such detoxification systems are of particular importance for methylotrophic microorganisms that rely on formaldehyde as a central metabolite in their one-carbon metabolism. Understanding formaldehyde dissimilation pathways in non-methylotrophic industrial microorganisms is necessary for ongoing research aiming at engineering methylotrophy into their metabolism (synthetic methylotrophy). There is a variety of formaldehyde dissimilation pathways across microorganisms, often based on the activity of formaldehyde dehydrogenases. In this study, we investigated the role of the *yycR* gene of *Bacillus subtilis* putatively encoding a novel, uncharacterized zinc-type alcohol dehydrogenase-like protein. We showed that the *B. subtilis* Δ*yycR* mutant displayed a reduced formaldehyde tolerance level and confirmed the enzymatic activity of recombinantly produced and purified YycR as formaldehyde dehydrogenase *in vitro*. Biochemical analyses demonstrated that YycR activity is optimal at 40°C, with the highest measured activity at pH 9.5, formaldehyde is the preferred substrate, and the kinetic constants are *K_m_* of 0.19 ± 0.05 mM and *V*_max_ of 2.24 ± 0.05 nmol min^−1^. Altogether, we showed that YycR is a novel formaldehyde dehydrogenase with a role in formaldehyde detoxification in *B. subtilis*, providing valuable insights for future research on synthetic methylotrophy in this organism.

**IMPORTANCE:**

Formaldehyde is a key metabolite in methanol assimilation for many methylotrophic microorganisms, and at the same time, it is toxic to all living cells, which means its intracellular concentrations must be tightly controlled. An in-depth understanding of methanol detoxification systems in industrially relevant microorganisms is a prerequisite for the introduction of methanol utilization pathways into their metabolism (synthetic methylotrophy). *Bacillus subtilis*, an industrial workhorse conventionally used for the production of enzymes, is known to possess two formaldehyde detoxification pathways. Here, we identify a novel formaldehyde dehydrogenase in this bacterium as a path towards creating innovative prospect strategies for strain engineering towards synthetic methylotrophy.

## INTRODUCTION

Formaldehyde is a prevalent environmental pollutant that can be generated by microorganisms as part of their metabolism (1–5). In methylotrophic bacteria and yeast, formaldehyde is an intermediate of one-carbon metabolism, while in non-methylotrophic microorganisms, it is a by-product of degradation of various raw materials, for example, lignin or choline (3–5). In some bacterial species, the methyl groups of glycine betaine are oxidized by *N*,*N*-dimethylglycine and sarcosine dehydrogenases (or oxidases) to form formaldehyde (6–8). Formaldehyde can also be created during Strecker degradation of glycine (9).

Despite being a fairly common intermediate of microbial metabolism and a prevalent air pollutant, formaldehyde is a potent electrophile that can react with biological nucleophiles in proteins and DNA and/or form dihydroxydimethyl peroxides or free radicals in the presence of oxidizing molecules, leading to oxidative stress and cell death (10–13). Formaldehyde is known to cause the formation of intra- and interstrand DNA cross-links and DNA-protein adducts and cross-links, which can be detrimental to cell fitness (13–17). As a result, all living organisms have developed formaldehyde detoxification processes, either through its oxidation to carbon dioxide or by condensation with metabolic intermediates (18–21).

Formaldehyde plays a crucial role in the assimilation of one-carbon compounds in methylotrophic organisms, and its intracellular concentration needs to be maintained below cell-toxic levels. This means that in instances where the intracellular formaldehyde content exceeds a toxic threshold, formaldehyde is inevitably subjected to dissimilation. Understanding formaldehyde metabolism in non-methylotrophic bacteria is of growing interest due to ongoing research to engineer methylotrophy (synthetic methylotrophy). A common approach supporting the introduction of synthetic methylotrophy in various bacterial hosts, such as *Corynebacterium glutamicum*, *Escherichia coli,* and *Bacillus subtilis,* has been the deletion of the formaldehyde dissimilatory pathways to avoid carbon escape through the oxidation of formaldehyde to CO_2_ (21–25).

Vegetative cells of wild-type *B. subtilis* have been reported to tolerate up to 2 mM formaldehyde, and *B. subtilis* spores exhibit up to 10% survival rate during exposure to 850 mM formaldehyde for 20 min (26, 27). To date, two formaldehyde detoxification pathways have been described in *B. subtilis*. One relies on the activities of 3-hexulose-6-phosphate synthase (Hps), which catalyzes the condensation of formaldehyde and ribulose 5-phosphate to hexulose 6-phosphate (Hu6P) and 6-phospho-3-hexuloisomerase (Phi), catalyzing the further isomerization of Hu6P into fructose 6-phosphate. The second pathway uses the activity of a bacillithiol-dependent aldehyde dehydrogenase AdhA and a DJ-1/PfpI-family cysteine proteinase (YraA). The *adhA–yraA* operon and the *yraC* gene, encoding a γ-carboxymuconolactone decarboxylase involved in the dissimilation of an aromatic compound, are positively regulated by the MerR-family regulator, YraB (AdhR) (26, 28, 29). The roles of the *hps-phi* operon and *adhA* gene in formaldehyde dissimilation have been experimentally confirmed, showing that both formaldehyde detoxification pathways are induced by exposure to formaldehyde, with HxlR and AdhR serving as transcriptional regulators, respectively (26, 30). Based on the reported existence of thiol-independent formaldehyde dehydrogenases in various *Pseudomonas* species, we were inspired to investigate whether a similar enzyme exists in *B. subtilis* (31–33).

Here, we describe the identification and characterization of a novel formaldehyde dehydrogenase encoded by the *yycR* gene in *B. subtilis*, which encodes an active formaldehyde dehydrogenase involved in formaldehyde detoxification in this bacterium.

## MATERIALS AND METHODS

### Strains, plasmids, and primers

*B. subtilis* 168, and the erythromycin-resistant deletion strains *B. subtilis* 168 *trcp2* Δ*adhA::erm* (reference number: BKE27010) and *B. subtilis* 168 *trcp2* Δ*yycR::erm* (reference number: BKE40250) were used for growth experiments. The deletion strains were purchased from the Bacillus Genetic Stock Center (BGSC). *E. coli* T7 Express was used as the expression host, *E. coli* DH5α was used as the cloning host, and *B. subtilis* 168 was the source of genomic DNA for cloning the *yycR* (locus tag: BSU_40250) and *adhA* (locus tag: BSU_27010) genes. Plasmids and bacterial strains used in this study are listed in Table 1, while primers used are tabulated in Table 2.

**TABLE 1:** Plasmids and bacterial strains used in this study

Name/abbreviated name	Relevant characteristics	Source
Plasmids		
pNIC-CH-CBM5	KanR; pNIC-CH derivative for the expression of a family 5 carbohydrate-binding module from *Cellvibrio japnonicus*	(34)
pNIC-CH-*yycR*	KanR; pNIC-CH derivative for the expression of *yycR* derived from *B. subtilis* 168	This study
pUB110Sxp	KanR; rolling circle-replicating, high-copy number plasmid for gene expression under the control of the inducible xylose promoter from *Bacillus megaterium*	(35)
pUB110Smp	KanR; rolling circle-replicating, high-copy number plasmid that contains the strong *mdh* promoter for the expression of gene of interest	(36)
pUB110Smp-*adhA*	KanR; pUB110Smp derivative for the expression of *adhA* derived from *B. subtilis* 168	This study
pUB110Smp-*yycR*	KanR; pUB110Smp derivative for the expression of *yycR* derived from *B. subtilis* 168	This study
Strains		
*E. coli* DH5α	F-*thi*-1 *end*A1 *hsd*R17(r-,m-) *sup*E44 _*lacU*169 (_80*lacZ*_M15) *recA*1 *gyrA*96 *relA*1	Stratagene
*E. coli* T7 Express	*fhuA2 lacZ:T7 gene1 [lon] ompT gal sulA11 R(mcr-73::miniTn10--Tet^S^)2 [dcm] R(zgb-210::Tn10--Tet^S^) endA1 Δ(mcrC-mrr)114::IS10*	New England Biolabs C2566
*E. coli* T7 Express (pNIC-CH-CMB5)	*E. coli* T7 Express strain for the expression of a family 5 carbohydrate-binding module from *Cellvibrio japnonicus*	NTNU strain collection
*E. coli* T7 Express (pNIC-CH-*yycR*)	*E. coli* T7 Express strain for the expression of *yycR* derived from *B. subtilis* 168	This study
*B. subtilis* wt	*B. subtilis* 168, wild-type strain	DSMZ 23778
*B. subtilis* Δ*adhA*	*B. subtilis* 168 *trcp2* Δ*adhA*::*erm* (reference number: BKE27010)	BGSC
*B. subtilis* Δ*yycR*	*B. subtilis* 168 *trcp2* Δ*yycR*::*erm* (reference number: BKE40250)	BGSC
*B. subtilis* Δ*adhA* (ev)	*B. subtilis* 168 *trcp2* Δ*adhA*::*erm* (pUB110Sxp); pUB110Sxp is an empty vector, referred to as ev	This study
*B. subtilis* Δ*yycR* (ev)	*B. subtilis* 168 *trcp2* Δ*yycR*::*erm* (pUB110Sxp); pUB110Sxp is an empty vector, referred to as ev	This study
*B. subtilis* Δ*adhA* (*adhA*)	*B. subtilis* 168 *trcp2* Δ*adhA*::*erm* (pUB110Smp-*adhA*)	This study
*B. subtilis* Δ*yycR* (*yycR*)	*B. subtilis* 168 *trcp2* Δ*yycR*::*erm* (pUB110Smp-*yycR*)	This study

**TABLE 2:** Primers used in this study

Primer name	Sequence 5′ → 3′	Function
FW_pNIC	CACCATCATCACCACCATTGA	Backbone of pNIC-CH-CBM5; forward primer
RV_pNIC	AGTATATCTCCTTCTTAAGGTTAAAC	Backbone of pNIC-CH-CBM5; reverse primer
FW_yycR	TTAAGAAGGAGATATACTATGGGAGGAATGGCGTTGACAG	*yycR* for pNIC-CH; forward primer
RV_yycR-2	AATGGTGGTGATGATGGTGCGCCTTCAATGTCCCATGCGGGTCGATG	*yycR* for pNIC-CH; reverse primer
FW_pLIC	TGTGAGCGGATAACAATTCC	Sequencing of pNIC-CH-*yycR,* forward primer
RV_pLIC	AGCAGCCAACTCAGCTTCC	Sequencing of pNIC-CH-*yycR,* reverse primer
FW_pUB110Smp_adhA	TACATAAATAGGAGGTAGTATCAATGTGTAATCAACATCAAACCCGTGTATTAAG	*adhA* for pUB110Smp, forward primer
RV_pUB110Smp_adhA	TGGCGGGTACCATATGGATCTTACAATGTGGAAATATCAATCACAAATCG	*adhA* for pUB110Smp, reverse primer
FW_pUB110Smp_yycR	TACATAAATAGGAGGTAGTATCAATGGGAGGAATGGCGTTGACAGGAAACA	*yycR* for pUB110Smp, forward primer
RV_pUB110Smp_yycR	TGGCGGGTACCATATGGATCTTACTTCAATGTCCCATGCGGGTCGATGAC	*yycR* for pUB110Smp, reverse primer
VPJF	TCTAATCCTTCTAAAAAATATAATTTAGAAAACTAAG	Sequencing of pUB110Smp derivatives*,* forward primer
VPJR	GGTGCGGGCCTCTTCGCTATTACG	Sequencing of pUB110Smp derivatives*,* reverse primer

### Media and growth conditions

To test the effect of formaldehyde on cell growth, *B. subtilis* 168 strains were cultivated in Luria-Bertani (LB) liquid medium supplemented with 0–1 mM formaldehyde. The following strains were used: *B. subtilis* 168 (the wild-type strain, hereafter referred to as wt), *B. subtilis* 168 *trcp2* Δ*adhA::erm* (referred to as Δ*adhA*), *B. subtilis* 168 *trcp2* Δ*yycR::erm* (referred to as Δ*yycR*), the empty vector strains *B. subtilis* 168 *trcp2* Δ*adhA::erm* (pUB110Sxp) [referred to as Δ*adhA* (ev)] and *B. subtilis* 168 *trcp2* Δ*yycR::erm* (pUB110Sxp) [referred to as Δ*yycR* (ev)], and the complementation strains *B. subtilis* 168 *trcp2* Δ*adhA::erm* (pUB110Sxp-*adhA*) [referred to as Δ*adhA* (*adhA*)] and *B. subtilis* 168 *trcp2* Δ*yycR::erm* (pUB110Sxp-*yycR*) [referred to as Δ*yycR* (*yycR*)]. Two consecutive precultures were cultivated in 25 mL of LB medium in shaking flasks, whereof second precultures were used as inoculum for the main cultures at an initial OD_600_ of 1.5–2. The media for the *B. subtilis* Δ*adhA* and Δ*yycR* strains were supplemented with 1 µg mL^−1^ of erythromycin, and for *B. subtilis* Δ*adhA* (ev), Δ*yycR* (ev), Δ*adhA* (*adhA*), and Δ*yycR* (*yycR*) strains, the media were supplemented with both 1 µg mL^−1^ of erythromycin and 25 µg mL^−1^ of kanamycin. All cultures were cultivated at 37°C and 200 rpm in biological triplicates. Cell growth was determined by measuring the OD_600_ value with a cell density meter (WPA CO 8000 Biowave). For inoculation, the second precultures were centrifugated for 5 min and 7,745 *g* at room temperature by using a 5430R Centrifuge (Eppendorf). After centrifugation, the supernatant was discarded, and each cell pellet was resuspended in 4 mL of LB medium with either 0, 0.5, or 1 mM formaldehyde and additional antibiotics. Subsequently, 1 mL of each resuspended cell solution was aliquoted in triplicates into BioLector (m2p-labs GmbH) flower plate. The fermentation was started in the BioLector microbioreactor (m2p-labs GmbH) at 37°C, 1,200 rpm, a humidity of 85%, a cycle time of 10, and a biomass gain of 7. Cell biomass formation based on scattered light was monitored for 24 h. Two-tailed Student’s *t*-test was used to compare control conditions (no formaldehyde supplementation) to each of the test conditions (0.5 or 1 mM formaldehyde supplementation).

For recombinant protein production, *E. coli* T7 Express (pNIC-CH-*yycR*) and the control strain *E. coli* T7 Express (pNIC-CH-CBM5) were inoculated each in 10 mL of LB supplemented with 50 µg L^−1^ of kanamycin and incubated overnight at 37°C (34). After overnight incubation, 5 mL of the overnight culture was added to two 500-mL flasks containing LB supplemented with 50 µg L^−1^ of kanamycin and incubated at 37°C and 225 rpm until an OD_600_ of 0.6–0.8 was reached (2–3 h). The cultures were induced with 0.1 mM isopropyl-β-D-thiogalactopyranoside (IPTG) and then incubated overnight at 22°C and 225 rpm. Cells were harvested by centrifugating the overnight cultures for 5 min at 4°C and 5,500 *g,* and the cell pellets were stored at -20°C.

### Molecular cloning

Competent *E. coli* T7 Express and *E. coli* DH5α cells were prepared according to Green and Rogers (37). All standard molecular cloning procedures were carried out as described in Sambrook and Russell (38) or according to the manuals provided by the manufacturers. PCR amplification was performed using the CloneAmp HiFi PCR Premix (Takara). Agarose gels 0.8% SeaKem LE (Lonza) were utilized to visualize the separation of DNA fragments. DNA fragments were isolated using QIAquick Gel Extraction Kit (Qiagen), and the Monarch Plasmid Miniprep Kit (New England Biolabs, T1010L) was used for plasmid DNA isolation.

For the construction of *E. coli* T7 Express (pNIC-CH-*yycR*) strain, the *yycR*-coding sequence was PCR amplified from the genomic DNA of *B. subtilis* wt, which was isolated according to the protocol described in Eikmanns et al. (39). To achieve the linearized pNIC-CH expression vector, the backbone of the pNIC-CH-CMB5 plasmid was PCR amplified and then digested with restriction enzyme DpnI (New England Biolabs). The digested pNIC-CH expression vector was purified and joined with the PCR-amplified *yycR* gene using Gibson assembly, creating the plasmid pNIC-CH-*yycR* (40). Subsequently, a heat shock transformation into *E. coli* T7 Express was performed (38). To create the complementation plasmids, the genes *adhA* and *yycR* were PCR amplified from the genomic DNA of *B. subtilis* wt isolated as described previously (39). Next, the pUB110Smp vector was digested with restriction enzymes BamHI and PciI (New England Biolabs). Each amplified gene and linearized plasmid DNA ends were joined with Gibson assembly (40). The resulting plasmids pUB110Smp-*adhA* and pUB110Smp-*yycR* were then transformed into *E. coli* DH5α via heat shock transformation (38). For colony PCR, GoTaq DNA Polymerase (Promega) was used. All sequences of cloned DNA fragments were verified and confirmed via Sanger sequencing (Eurofins Genomics). *B. subtilis* strains were transformed using the high salt/low salt transformation method (41). To establish the *B. subtilis* Δ*adhA* (ev) and Δ*adhA* (*adhA*) strains, the *B. subtilis* Δ*adhA* strain was transformed with the plasmids pUB11Sxp and pUB110Smp-*adhA*, respectively. To establish *B. subtilis* Δ*yycR* (ev) and Δ*yycR* (*yycR*) strains, the strain *B. subtilis* Δ*yycR* was transformed with the plasmids pUB110Sxp and pUB110Smp-*yycR*, respectively.

### Phylogenetic tree

Neighbor-joining tree analysis was conducted using BLOSUM62 (42). BLOSUM62 uses one of the available substitution matrices to compute a sum of scores for the residue pairs at each aligned position to compute the distances between individual enzymes. Phylogenetic trees are calculated on the basis of a measure of similarity between each pair of sequences in the alignment and visualized using software Jalview (http://www.jalview.org).

### Detection of signature matches in YycR sequence with ScanProsite

ScanProsite web interface (https://prosite.expasy.org/scanprosite/) was used to scan the YycR sequence against a PROSITE protein motif database (43, 44).

### Multiple sequence alignment with Clustal Omega

The multiple sequence alignment was conducted with Clustal Omega (https://www.ebi.ac.uk/Tools/msa/), and the figure was generated using Jalview (45, 46). Clustal Omega uses seeded guide trees and Hidden Markov model (HMM) profile-profile techniques to generate alignments between three or more sequences.

### Protein 3D structure prediction with AlphaFold

The 3D coordinates of YycR in complex with Zn^2+^ and NAD^+^ were generated using AlphaFold 3 using the web interface available at https://alphafoldserver.com/ (47). The inputs were three molecule types: the protein was defined using the primary sequence of YycR (UniProt Q45604), the two ions were defined as Zn^2+^, and NAD^+^ as the ligand. Protein structures were visualized in PyMOL. The predicted YycR 3D structure was aligned with the crystal structure of *Pseudomonas putida* formaldehyde dehydrogenase (FDH) (PDB ID: 1KOL_A) using the combinatorial extension algorithm, which uses several aligned fragment pairs (pairs of fragments from both proteins that have similar local structure) to generate an optimal global alignment of the two proteins (48).

### Recombinant protein production and purification

The *E. coli* T7 Express (pNIC-CH-*yycR*) and the control strain *E. coli* T7 Express (pNIC-CH-CBM5) cell pellets were thawed on ice and gently resuspended in 20 mL of lysis buffer (20 mM Tris-HCl, 500 mM NaCl, and 0.05% Triton X-100) supplemented with an ethylenediaminetetraacetic acid (EDTA)-free complete ultra protease inhibitor tablet (Roche). This was followed by 5 min of sonication (40% amplitude, pulse on 2 s, pulse off 2 s), using a Fisherbrand Model 505 Sonic Dismembrator equipped with a microtip. The cell lysates were centrifuged for 20 min at 4°C and 20,000 *g* and then supernatants were filtered using a syringe and a 0.25 µm pore size filter. Prior to protein purification, 1 mL of Buffer B (50 mM Tris-HCl, 300 mM NaCl, and 400 mM imidazole, pH 8) was added to the filtered cell lysate.

Protein purification was performed on an ÄKTApure fast protein liquid chromatography (FPLC) system (Cytiva). All FPLC steps were performed with a flow rate of 5 mL min^−1^. First, the crude extract was loaded onto a 5 mL HisTrapHP column (Cytiva) that had been equilibrated using a mixture of 95% Buffer A (50 mM Tris-HCl and 300 mM NaCl pH 8) and 5% Buffer B. Unbound proteins (i.e., flow through) were washed from the column by using a linear gradient of 5%–25% Buffer B over 22 column volumes. YycR was eluted by increasing the concentration of Buffer B to 50%. Fractions FT (flow through), F1 (YycR), and F2 (YycR) were collected and analyzed by sodium dodecyl sulfate-polyacrylamide gel electrophoresis (SDS-PAGE) together with the crude extracts of strains *E. coli* T7 Express (pNIC-CH-CBM5) and *E. coli* T7 Express (pNIC-CH-*yycR*). Gels were run under denaturing conditions using SurePAGE BISTRIS gels (GenScript) and MES-SDS running buffer (GenScript), followed by staining using the eStain L1 Protein Staining System (GenScript). PAGE-MASTER Protein Standard Plus (GenScript) was used to estimate the molecular weight of target proteins. The molecular weight of YycR (MW: 44 kDa) and the theoretical extinction coefficient for YycR (*ε* = 27,890 M^−1^ cm^−1^) were estimated from its amino acid sequence by the ExPasy ProtParam server (https://web.expasy.org/protparam/) (49). The protein concentration was determined using the theoretical extinction coefficient of YycR and its absorbance at a wavelength of 280 nm (*A*_280_) measured with Nanodrop. A protein yield of 20 mg per liter of cell culture was calculated.

### Formaldehyde dehydrogenase activity assay

Enzymatic activity of YycR was assayed using an Infinite M200 Pro-Microplate reader (TECAN), following the formation of NADH at 340 nm (Δ_*ε*_ = 6,230 M^−1^ cm^−1^) for 8 min at 40°C, based on the formaldehyde dehydrogenase activity assay described by Lessmeier et al. (50) with some modifications. Triplicate measurements were performed in 96-well plates, with a final reaction volume of 300 µL. The reaction mixtures contained 50 mM BISTRIS propane (pH 9.0), 25 mM NaCl, 10 mM NAD^+^, and 240 nM (0.0106 mg mL^−1^) YycR. These were equilibrated to the desired reaction temperature for 4 min in the spectrophotometer before 1 mM formaldehyde was added to start the reaction. The specific activity of YycR (U mg^−1^) was calculated from the absorption (Abs min^−1^) of the reaction mixtures corrected by subtracting the baseline absorption (Abs min^−1^) of the reaction mixtures without added substrate.

The optimal pH of the reaction was determined by performing the enzymatic activity assay as described above using 50 mM MES with 25 mM NaCl (pH 5.5, 6.0, and 6.5) or 50 mM BISTRIS propane with 25 mM NaCl (pH 6.5, 7.0, 7.5, 8.0, 8.5, 9.0, and 9.5) as buffers. The optimal reaction temperature was determined by setting the reaction temperature to 30°C, 35°C, 37°C, 40°C, and 42°C using Zn^2+^ as a cofactor. The thermal stability of YycR was assayed by pre-incubating the reaction mixtures (without formaldehyde and NAD^+^) at 20°C, 30°C, 40°C, 50°C, 60°C, and 70°C for 1 h before starting the reactions. The mixtures were cooled to room temperature, 10 mM NAD^+^ was added, and the assay was performed as described above, with a reaction temperature of 40°C. The effect of adding different metal ions was assayed by employing a metal-binding procedure modified from reference (51) before carrying out the assays. The enzyme (0.6 µM) was incubated with a threefold molar excess (i.e., 1.8 µM) of MgCl_2_, CaCl_2_, MnCl_2_, CuCl_2_, CoCl_2_, NiCl_2_, FeSO_4_, or ZnCl_2_ in 50 mM BISTRIS propane (pH 9.0), 25 mM NaCl for 1 h at 20°C with 400 rpm mixing. The excess metal ions were removed by passing 1 mL of the solution through a PD MidiTrap G-25 desalting column, collecting only the first 1 mL of the eluate. A control sample was made to account for any pre-bound metals present in the purified enzyme by incubating the enzyme with 1 mM EDTA in 50 mM BISTRIS propane (pH 9.0) and 25 mM NaCl for 1 h at 20°C with 400 rpm mixing to extract bound metal ions. Excess EDTA was removed by desalting, following the same procedure as for the metal salts. The pre-treated YycR-metal solutions were assayed as described earlier, with a reaction temperature of 37°C. The substrate preference of YycR was assayed by adding 1 mM of ethanol, methanol, 1-butanol, formic acid, glyoxal, and acetaldehyde instead of formaldehyde to start the reaction. To estimate Michaelis-Menten parameters, the enzymatic assays were performed at formaldehyde concentrations in the range of 0.6–40 mM. The *K_m_* (mM) and *V*_max_ (nmol min^−1^) were estimated by fitting the measured data points to the Michaelis-Menten equation *v_0_ = V*_max_[S]/(*K*_m_ *+* [S]), where S represents formaldehyde. The fitting was done by using the scipy.optimize.curve_fit function in Python, and the errors (standard deviation) were determined from the diagonal of the estimated covariance matrix of the parameters.

### NMR spectroscopy

The ^1^H-^13^C HSQC spectrum of the reaction mixture (500 mM YycR, 10 mM NAD^+^, 25 mM NaCl, and 30 mM formaldehyde in 50 mM BISTRIS propane, pH 9.0, supplemented with 10% D_2_O) was recorded on a Bruker Ascend 800-MHz spectrometer Avance III HD equipped with a 5-mm Z-gradient CP-TCI (H/C/N) cryoprobe at the NV-NMR Center/Norwegian NMR Platform in Trondheim, Norway. The mixture was prepared 4 h before recording the spectrum. The spectrum was processed and visualized using MestReNova version 14.1.2.

## RESULTS AND DISCUSSION

### *In silico* analysis of *B. subtilis* genome sequence identified a putative novel formaldehyde dehydrogenase gene *yycR*

**Fig 1:**
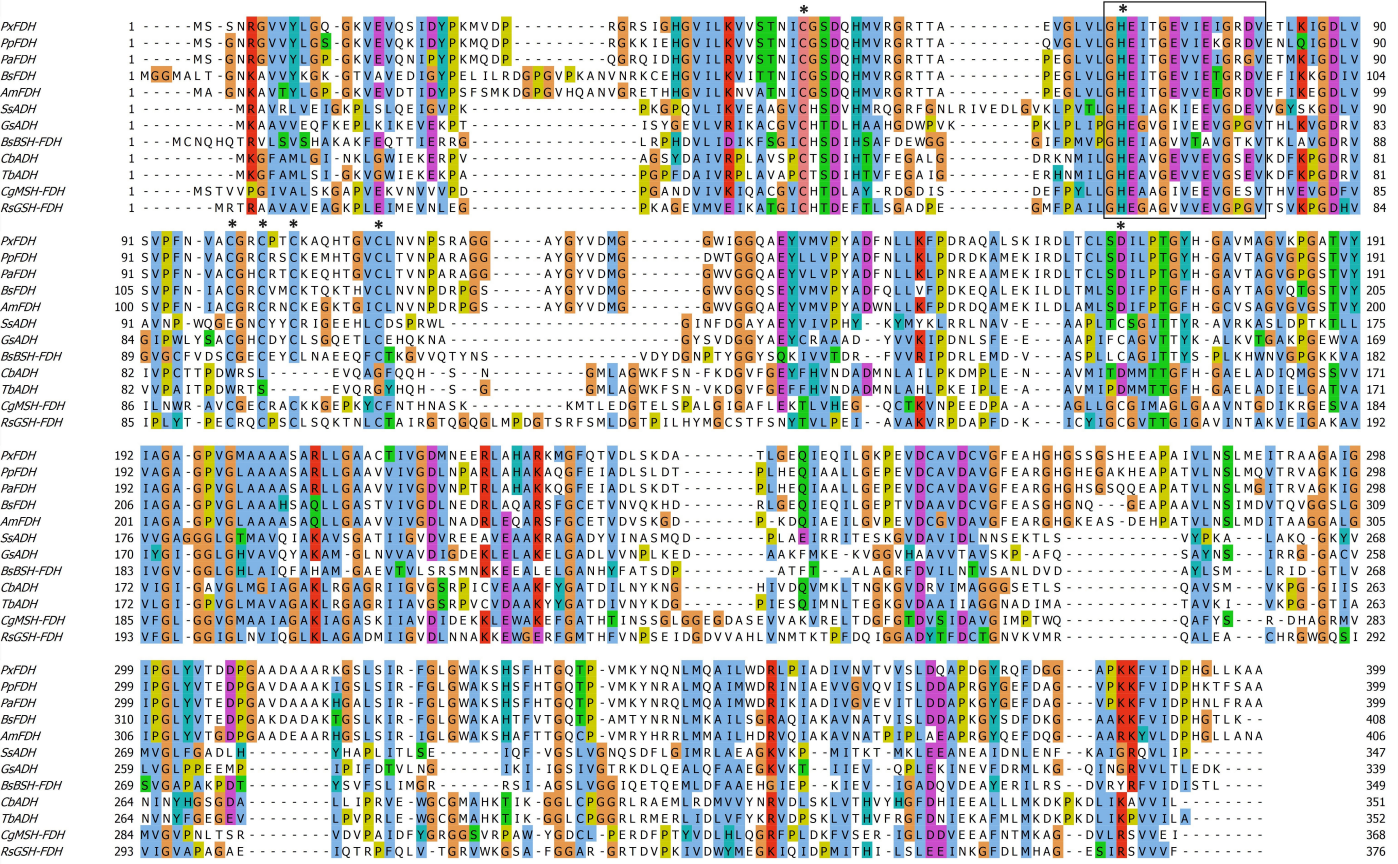
Primary sequence alignment of alcohol dehydrogenases (ADHs) and formaldehyde dehydrogenases. The amino acid sequences were retrieved from GenBank and aligned using Clustal Omega (46). The box indicates the putative zinc-containing alcohol dehydrogenases signature sequence (GHEITGEVIETGRDV) detected by ScanProsite based on BsFDH sequence (43). Starred amino acids indicate the residues involved in Zn^2+^ binding as characterized in FDH from *P. putida* (55). Amino acid residues are numbered on the right and left. The figure was generated using Jalview (45). PxFDH, Fdh from *P. xenovorans* (GenBank ID: AIP37160) (4); PpFDH, Fdh from *P. putida* (GenBank ID: D21201) (56); PaFDH, Fdh from *P. aeruginosa* (GenBank ID: Q9HTE3) (54). AmFDH, Fdh from *A. methanolica* (GenBank ID: AIJ21411) (53); BsFDH, YycR from *B. subtilis* (GenBank ID: NP_391905); SsADH, Ahd from *Saccharolobus solfataricus* (GenBank ID: P39462) (57); GsAHD, Adh from *Geobacillus stearothermophilus* (GenBank ID: P42328) (58); BsBSH-FDH, bacilithiol-dependent Fdh from *B. subtilis* (GenBank ID: NP_390579) (26); CbADH, Adh from *Clostridium beijerinckii* (GenBank ID: P25984) (59); TbADH, Adh from *Thermoanaerobacter brockii* (GenBank ID: P14941) (59); CgMSH-FDH, mycothiol-dependent Fdh from *Corynebacterium glutamicum* (GenBank ID: CAF18890) (50); RsGSH-FDH, glutathione-dependent Fdh from *Cereibacter sphaeroides* (GenBank ID: AAB09774) (60). The amino acids were assigned colors according to Clustal X color scheme. Each residue in the alignment is assigned a color if the amino acid profile of the alignment at that position meets some minimum criteria specific for the residue type.

Previous studies have established that AdhA is involved in the bacillithiol-dependent linear formaldehyde dissimilation pathway in *B. subtilis* and that the activities of Hps and Phi are part of formaldehyde detoxification metabolism (26, 28, 29). Here, we performed the protein BLASTP analysis of the protein database of *B. subtilis* 168 using the *Pseudomonas putida*-derived thiol-independent formaldehyde dehydrogenase as a protein query sequence (GenBank ID: D21201) (32, 52). We identified YycR as an uncharacterized enzyme that putatively belongs to the zinc-containing alcohol dehydrogenase family. The YycR primary sequence displayed high similarities to the glutathione-independent formaldehyde dehydrogenases from *Amycolatopsis methanolica*, *P. putida*, *Pseudomonas aeruginosa,* and *Paraburkholderia xenovorans* (Fig. 1; Fig. S1) (4, 32, 53, 54). Thiol-independent formaldehyde dehydrogenases are rare, and the catalytic mechanism of these enzymes is still unknown (55). Further analysis using ScanProsite, which searches for hits by specific motifs in protein sequence databases, revealed the presence of a zinc-containing alcohol dehydrogenase signature sequence in YycR (43). We used AlphaFold 3 to predict the structure of YycR in complex with the cofactors NAD^+^ and Zn^2+^ (Fig. S2) and aligned the structure to the crystal structure of formaldehyde dehydrogenase from *P. putida* (32). The two structures are almost identical, with a root mean square deviation of only 1.01 Å. Both enzymes bind two Zn^2+^ ions, one in the active site and one with a structure-stabilizing function (56).

The predicted YycR structure (Fig. S2) indicates coordination of the catalytic Zn^2+^ by C61, H82, and D184 and the structural Zn^2+^ by the four cysteine residues C112, C115, C118, and C126. These residues correspond to the characterized zinc-binding residues in *P. putida* formaldehyde dehydrogenase (55), which are starred in the sequence alignment shown in Fig. 1. Similarly to the formaldehyde dehydrogenase in *P. putida*, YycR contains a zinc-binding aspartate residue (D184), which is replaced by a cysteine in some of the other formaldehyde and alcohol dehydrogenases (Fig. 1).

### *B. subtilis* Δ*yycR* displayed reduced tolerance to formaldehyde in shake flask cultivations

**Fig 2:**
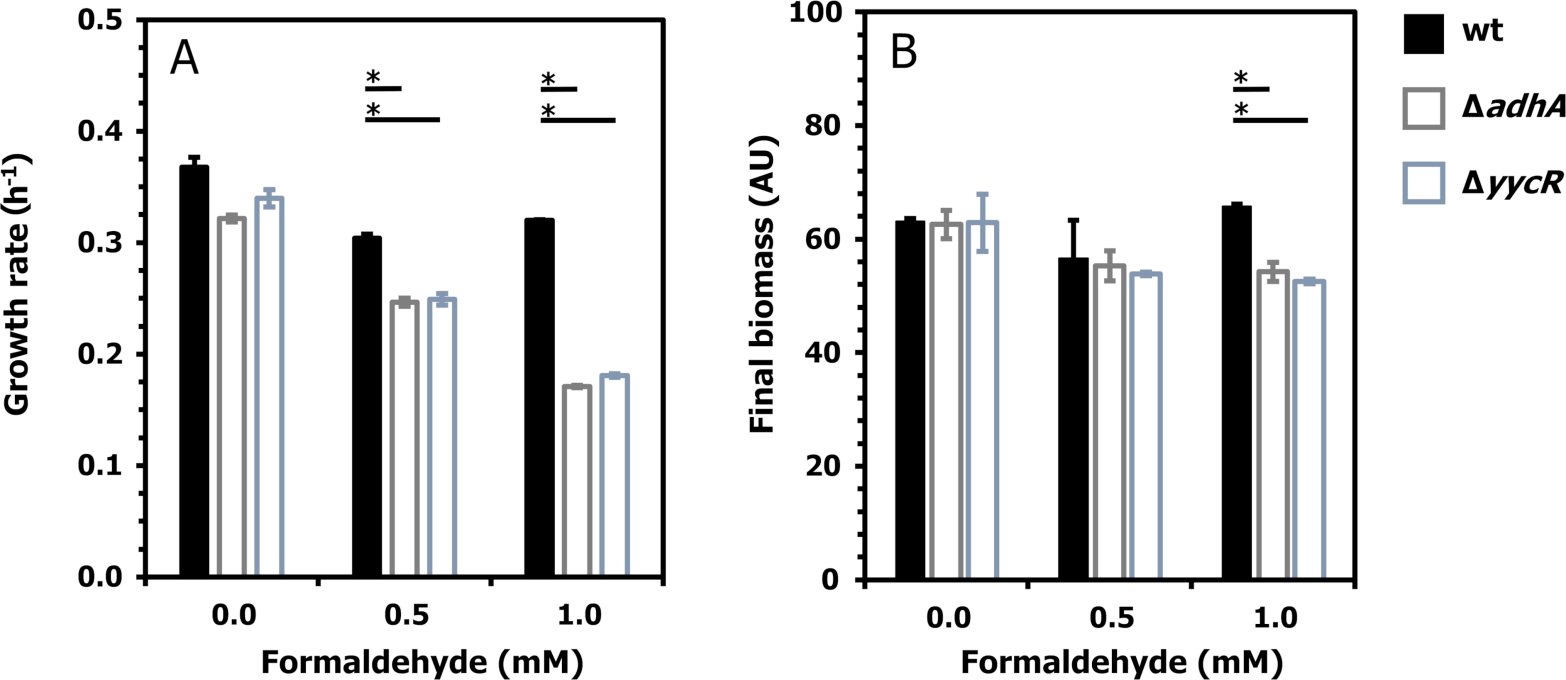
Effect of formaldehyde on the growth of *B. subtilis* wt and Δ*adhA and* Δ*yycR* strains. The *B. subtilis* deletion strains Δ*adhA and* Δ*yycR* and the control strain *B. subtilis* 168 wt were cultivated in LB broth supplemented with different concentrations of formaldehyde. Cultivation was performed in the BioLector, and cell growth was monitored for 24 h, which is represented as growth rate in h^−1^ (**A**) and biomass formation in arbitrary units (AU) (**B**). The results are presented as mean values of biological triplicates with standard deviations. The horizontal lines with an asterisk (*) link the values significantly different from the value of the corresponding control (0 mM formaldehyde) according to *t*-test, *P* < 0.001, *n* = 3.

To test our hypothesis that *yycR* encodes formaldehyde dehydrogenase activity, we first cultivated *B. subtilis* wt, *B. subtilis* Δ*adhA,* and *B. subtilis* Δ*yycR* (see descriptions in Table 1) in LB medium supplemented with 0–1 mM of formaldehyde. Our results confirmed that *B. subtilis* wt can tolerate 1 mM formaldehyde without significant reduction in growth rate and that both *B. subtilis ΔadhA* and *B. subtilis* Δ*yycR* strains displayed significantly decreased growth rate (from 0.32 to 0.17 h^−1^ and from 0.34 to 0.18 h^−1^), respectively, in 1 mM formaldehyde (Fig. 2A) (26). For *B. subtilis ΔadhA,* these results are in agreement with previously published reports about the involvement of AdhA in formaldehyde detoxification (26), and they also indicate for the first time that the *yycR* gene is involved in formaldehyde detoxification in *B. subtilis*. The effect of formaldehyde on final biomass formation was only significant in the case of the *B. subtilis* Δ*yycR* strain, while the final biomass remained unchanged during growth in 1 mM formaldehyde in comparison to control conditions for the *B. subtilis ΔadhA* strain (Fig. 2B).

Our results suggest the existence of three parallel formaldehyde detoxification systems in *B. subtilis*. This is not an unusual occurrence for bacterial species that multiple formaldehyde detoxification systems are active in parallel; for example, *Burkholderia fungorum* LB400 possesses three formaldehyde detoxification systems, namely a NAD-dependent, thiol-independent formaldehyde dehydrogenase; a NAD- and thiol-dependent formaldehyde detoxification pathway; and a tetrahydromethanopterin-methanofuran-dependent formaldehyde detoxification pathway (4). While all three formaldehyde detoxification pathways are active in *B. fungorum*, they are at the same time functionally redundant (4). In *B. subtilis,* the expression of the condensation-dependent and thiol-dependent pathways is regulated by the respective regulatory proteins, HxlR and AdhR (26). The deletion of each of the pathways leads to growth deterioration in the presence of 1 mM formaldehyde, while the growth pattern of wild-type strain remains unaffected in this condition (26, 28). This suggests that the formaldehyde metabolism is intertwined between multiple detoxification systems in *B. subtilis*, highlighting the probability of the existence of a third detoxification pathway.

### Complementation with *yycR* gene partly restored formaldehyde tolerance in *B. subtilis* Δ*yycR*

**Fig 3:**
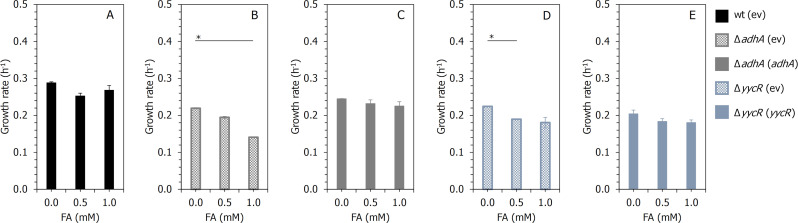
Effect of formaldehyde on the growth of *B. subtilis* wt (ev) (A), Δ*adhA* (ev) (B), Δ*yycR* (ev) (C), Δ*adhA* (*adhA*) (D), and Δ*yycR* (*yycR*) (E) strains. Recombinant *B. subtilis 168* strains were cultivated in LB broth supplemented with either 0, 0.5, or 1 mM of formaldehyde. The BioLector was used for cell cultivation and monitoring cell growth for 24 h. The results are depicted as growth rate in h^−1^ and presented as mean values of biological triplicates with standard deviations. The horizontal lines with an asterisk (*) link the values significantly different from the value of the corresponding control (0 mM formaldehyde) according to *t*-test, *P* < 0.001, *n* = 3.

As depicted in Fig. 2, the deletion of *adhA* or *yycR* reduces the tolerance of *B. subtilis* to formaldehyde. These two deletion strains were next transformed with the empty plasmid vector pUB110Sxp and with this plasmid vector carrying the respective *adhA* or *yycR* gene under the control of a methanol dehydrogenase promoter derived from *Bacillus methanolicus*, leading to the creation of following strains: *B. subtilis* Δ*adhA* (ev), *B. subtilis* Δ*adhA* (*adhA*), *B. subtilis* Δ*yycR* (ev), and *B. subtilis* Δ*yycR* (*yycR*) (see Table 1). We have previously confirmed that the *B. methanolicus*-derived methanol dehydrogenase promoter of expression vector pUB110Smp is functional in *B. subtilis* using sfGFP as a reporter protein (61). Transformation of *B. subtilis* wt with an empty vector did not have an effect on formaldehyde tolerance (Fig. 2A and Fig. 3A). The complementation of the *adhA* deletion by plasmid-based *adhA* expression in the *B. subtilis* Δ*adhA* (*adhA*) strain led to partial restoration of the formaldehyde tolerance level (Fig. 3B and C). *B. subtilis* Δ*adhA* (*adhA*) displayed an 8% reduced growth rate in the presence of 1 mM formaldehyde (Fig. 3C), while its control strain *B. subtilis* Δ*adhA* (ev) displayed a 36% reduced growth rate under these conditions (Fig. 3B). The control strain *B. subtilis* Δ*yycR* (ev) displayed a 20% reduced growth rate in the presence of 1 mM formaldehyde while *B. subtilis* Δ*yycR* (*yycR*) displayed a 12% reduced growth rate under such conditions (Fig. 3D and E). Although complementation of the two mutant strains did not completely restore the wild-type phenotype, these results support our hypothesis that *yycR*, together with *adhA*, plays a role in formaldehyde detoxification in *B. subtilis*.

### *In vitro* characterization of purified YycR confirms its formaldehyde dehydrogenase activity

Based on the results above indicating that *yycR* is involved in formaldehyde detoxification in *B. subtilis*, we conducted experiments to recombinantly produce, purify, and assess the enzymatic activity of YycR *in vitro*. The cell lysates of *E. coli* T7 Express (pNIC-CH-*yycR*) and the control strain *E. coli* T7 Express (pNIC-CH-CMB5) (Table 1) were subjected to affinity His-tagged YycR purification via FPLC (Fig. S3). Based on the predicted primary sequence of the *yycR* gene product using the Expasy ProtParam tool, the deduced molecular weight of YycR is 44 kDa, which is in accordance with the size of the bands from both fractions F1 and F2 (Fig. S4) (49). YycR has a similar molecular weight to the monomeric formaldehyde dehydrogenases from *Mycobacterium smegmatis*, *A. methanolica* and *P. putida* (32, 62, 63). Using the enzymatic assay with formaldehyde as a substrate, we confirmed that the purified YycR catalyzes formaldehyde oxidation (Table S1).

### Optimal enzymatic activity of YycR is observed at pH 9.5 and 40°C

**Fig 4:**
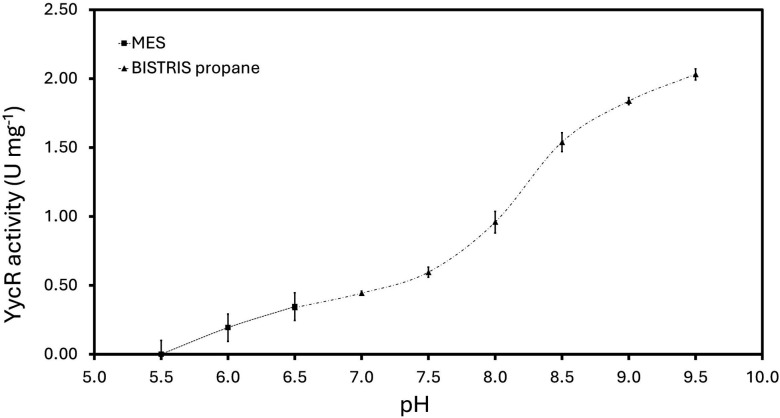
Specific activity of YycR at varying pH. Activity assays were performed with YycR (240 nM) in 50 mM MES or BISTRIS propane and 25 mM NaCl over a pH range of 5.5–9.5. The pH buffered with MES is depicted with squares, while BISTRIS propane is shown with triangles. Reactions were started by the addition of 1 mM formaldehyde to the reaction mixture, and the activity is reported as U mg^−1^. Triplicates are shown.

The biochemical properties of YycR *in vitro* were analyzed based on the *in silico* analysis, which predicted YycR to be a putative zinc-type alcohol dehydrogenase. We tested the enzymatic activity of the purified YycR in the pH range between 5.5 and 9.5 and with two different buffers, MES and BISTRIS propane, to ensure the necessary pH buffering capacity (Fig. 4). YycR was catalytically active at all tested pH values except for 5.5, and the highest activity of 2.03 ± 0.04 U mg^−1^ was obtained at a pH value of 9.5; however, higher pH values were not tested and we decided to perform all subsequent enzyme assays using BISTRIS propane buffer adjusted to pH 9 (Fig. 4). This result is similar to the optimal pH of 8.9 for a closely related thiol-independent formaldehyde dehydrogenase from *P. putida* (56). Both MES and BISTRIS propane were tested at pH 6.5, which is a threshold value for the buffering capacity of these buffers.

**Fig 5:**
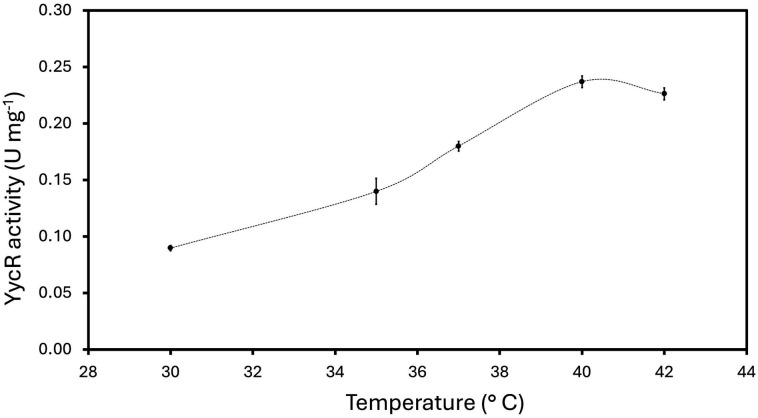
Specific activity of YycR at varying temperatures. Activity assays were performed with purified YycR (240 nM) using 50 mM BISTRIS propane, 25 mM NaCl, pH 9 as buffer. The enzymatic reaction was performed at increasing temperatures. Reactions were started by the addition of 1 mM formaldehyde to the reaction mixture and are reported as U mg^−1^. Triplicates are shown.

Next, we tested YycR enzymatic activity at different reaction temperatures ranging from 30°C to 42°C using the predicted Zn^2+^ cofactor (Fig. 5). The highest enzymatic activity of 0.24 ± 0.01 U mg^−1^ was observed at 40°C, which is almost triple the activity observed at 30°C.

The thermal stability of YycR was assayed by measuring the enzymatic activity after 1-h incubation at temperatures ranging from 30°C to 70°C. We observed that YycR is stable at 30°C for 1 h, with slightly lower activity at 40°C, whereas almost no activity could be measured after 1-h incubation at 50°C and above (Table 3).

**TABLE 3:** Thermal stability of YycR^*a*^

Incubation temperature (°C)	Specific activity (U mg^−1^)
30	0.25 ± 0.02
40	0.23 ± 0.01
50	0.07 ± 0.01
60	n.d.

^*a*^ Activity assay mixtures containing YycR (240 nM) in 50 mM BISTRIS propane and 25 mM NaCl, pH 9 were incubated for 1 h at increasing temperatures. The reactions were then started by the addition of 1 mM formaldehyde to the reaction mixture and were carried out at 37°C. n.d., no detected specific activity. Triplicates are shown.

Enzyme assays were performed using various divalent metal ions as cofactors. Our results show that neither incubation with EDTA nor additional supplementation with metal ions affect YycR activity (Table 4). These observations might lead to the erroneous conclusion that YycR is metal independent. However, it is more plausible that the catalytic metal ion is deeply embedded in the enzyme’s structure, as can be seen in the predicted structure of the active site of the YycR in complex with Zn^2+^ and NAD^+^ (Fig. S2). We hypothesize that due to this strong Zn^2+^ interaction, incubation with EDTA or with other metal ions may not be sufficient to displace the already bound metal.

**TABLE 4:** Effect of metal ions on the specific activity of YycR^*a*^

Ion	Specific activity (U mg^−1^)
As purified	0.21 ± 0.01
EDTA treated	0.24 ± 0.01
Zn^2+^	0.18 ± 0.01
Mg^2+^	0.19 ± 0.01
Ca^2+^	0.22 ± 0.02
Mn^2+^	0.21 ± 0.02
Cu^2+^	0.17 ± 0.01
Co^2+^	0.19 ± 0.02
Ni^2+^	0.20 ± 0.01
Fe^2+^	0.20 ± 0.01

^*a*^ Activity assays were performed at 37°C using YycR (240 nM) in 50 mM BISTRIS propane and 25 mM NaCl, pH 9, with different cations as cofactors. The reactions were started by adding 1 mM formaldehyde to the assay mix. Triplicates are shown.

### YycR catalyzes the conversion of formaldehyde to formic acid

**Fig 6:**
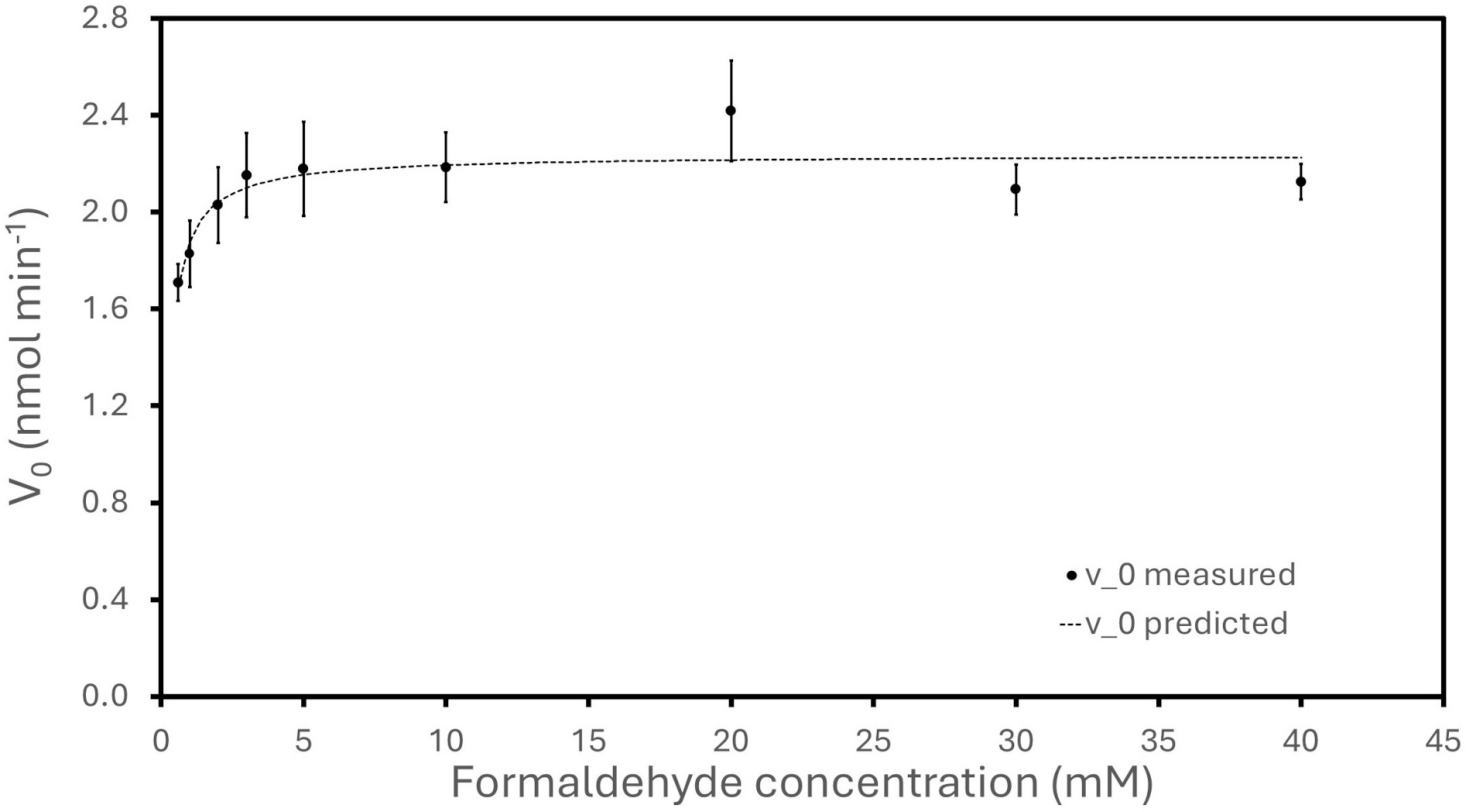
Michaelis-Menten plot of initial rate (nmol min^−1^) as a function of formaldehyde concentration (mM). The *K_m_* and *V*_max_ were estimated by solving for a minimal sum of square differences between experimental and theoretical data points obtained from the Michaelis-Menten formula. Enzymatic activity assays were performed at 37°C using purified YycR (240 nM) in 50 mM BISTRIS propane, 25 mM NaCl, pH 9. To start the reaction, different concentrations of formaldehyde were added. Triplicates are shown.

Based on the observation that YycR possesses an alcohol dehydrogenase domain, we tested methanol, ethanol, and 1-butanol as potential alternative substrates to formaldehyde. We also tested enzymatic activity on acetaldehyde, glyoxal, and formic acid. Low enzymatic activity was detected for acetaldehyde (about 10-fold lower than for formaldehyde), and no enzymatic activity could be detected for the other substrates (Table S2), indicating that formaldehyde is the preferred substrate for this enzyme under the *in vitro* conditions tested. Acetaldehyde contains an additional methyl group, which seems to hinder its access to the active site. To identify the products of the YycR-catalyzed activity on formaldehyde, we recorded a ^1^H-^13^C HSQC NMR spectrum (Fig. S5) of the YycR formaldehyde reaction mixture after 4 h of reaction time. Formic acid was detected with chemical shifts of 8.35 and 171.1 ppm in the ^1^H and ^13^C dimension, respectively. This confirms that YycR functions as a formaldehyde dehydrogenase, which is in agreement with the high similarity between our predicted YycR structure and the crystal structure of the previously characterized formaldehyde dehydrogenase from *P. putida* (32). The kinetic parameters for YycR with formaldehyde as the substrate were determined from a Michaelis-Menten assay (Fig. 6). The *K*_*m*_ and *V*_max_ were estimated to be 0.19 ± 0.05 mM and 2.24 ± 0.05 nmol min^−1^, respectively, which corresponds to a *k*_cat_ of 0.52 s^−1^ and a catalytic efficiency (*k*_cat_/*K*_*m*_) of 2.74 s^−1^ mM^−1^. Compared to the previously reported parameters of formaldehyde dehydrogenase from *P. putida*, *K*_*m*_ = 0.37 mM, *k*_cat_ = 33.3 s^−1^, and *k*_cat_/*K*_*m*_ = 90.0 s^−1^mM^−1^, YycR displays higher substrate affinity but significantly lower turnover at the conditions tested in each study (55). The biological impact of these different values is unknown and would be dependent on the homeostatic levels at which formaldehyde is regulated.

### Conclusion

In this paper, we have identified and characterized a novel formaldehyde dehydrogenase YycR in *B. subtilis*. Our results indicate that *B. subtilis* possesses one additional formaldehyde detoxification pathway, beyond the Hps-Phi and AdhA-mediated pathways, and its capability to sporulate and survive at comparatively high formaldehyde concentrations in this form. The newly discovered YycR is a thiol-independent enzyme that uses NAD^+^ and metal ions as cofactors. It appears to have a rather narrow substrate range, as only formaldehyde could serve as a substrate relative to numerous others tested. The affinity of this *B. subtilis* dehydrogenase for formaldehyde, with a *K_m_* of 0.19 ± 0.05 mM, is comparable to other thiol-independent formaldehyde dehydrogenases. These findings should be useful for future design aiming at engineering synthetic methylotrophy into *B. subtilis* or alternative hosts.

## AFTER EPUB

*[This article was published on 29 October 2024 with an error in the supplemental material file. The supplemental file was updated in the current version, posted on 8 November 2024.]*

## SUPPLEMENTAL MATERIAL

Supplemental material aem.02181-23-s0001.pdf Figures S1 to S5; Tables S1 and S2.

ASM does not own the copyrights to Supplemental Material that may be linked to, or accessed through, an article. The authors have granted ASM a non-exclusive, world-wide license to publish the Supplemental Material files. Please contact the corresponding author directly for reuse.
