# Supplementary material for: Identification and characterization of a novel formaldehyde dehydrogenase in Bacillus subtilis
Source: Appl Environ Microbiol. 2024 Oct 29;90(11):e02181-23. doi: 10.1128/aem.02181-23 (PMC11577753; doi:10.1128/aem.02181-23)
Supplement: Supplemental material — Figures S1 to S5; Tables S1 and S2. [file aem.02181-23-s0001.pdf]

Supplementary material

to

Identification and characterization of a novel formaldehyde dehydrogenase in *Bacillus subtilis*

Vivien Jessica Klein[1,§], Susanne Hansen Troøyen[1,§], Luciana Fernandes Brito[1], Gaston Courtade[1], Trygve Brautaset[1], Marta Irla[1,*,‡]

[1]Department of Biotechnology and Food Science, NTNU Norwegian University of Science and Technology, Trondheim, Norway

[§]V.J.K. and S.H.T. contributed equally to this work.

*Corresponding author: Marta Irla
Email: marta.irla@bce.au.dk

[‡] Current Address: Department of Biological and Chemical Engineering, Aarhus University, 8000 Aarhus, Denmark

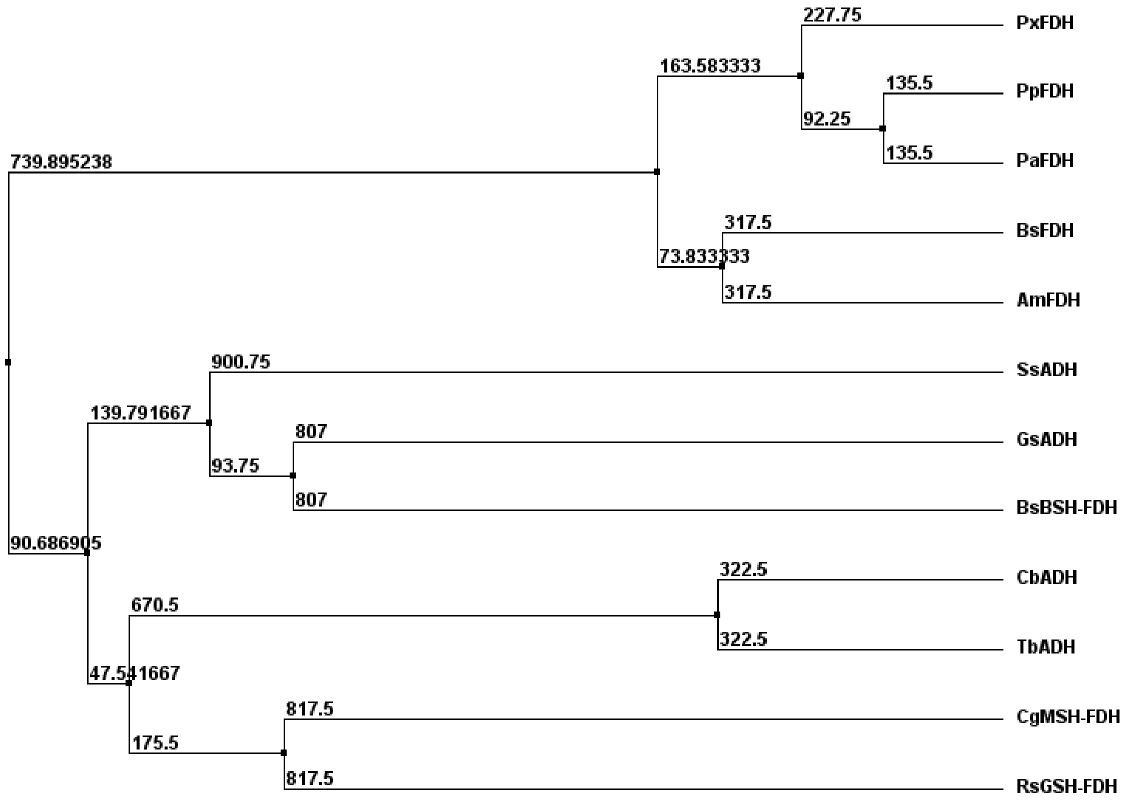

Figure S1: Neighbor joining tree using BLOSUM62 (1). BLOSUM62 uses one of the available substitution matrices to compute a sum of scores for the residue pairs at each aligned position. The distances between individual enzymes are shown (2). PxFDH, Fdh from *Paraburkholderia xenovorans* (GenBank ID: AIP37160) (3); PpFDH, Fdh from *Pseudomonas putida* (GenBank ID: D21201) (4); PaFDH, Fdh from *Pseudomonas aeruginosa* (GenBank ID: Q9HTE3) (5). AmFDH, Fdh from *Amycolatopsis methanolica* (GenBank ID: AIJ21411) (6); BsFHD, YycR from *Bacillus subtilis* (GenBank ID: NP_391905); SsADH, Ahd from *Saccharolobus solfataricus* (GenBank ID: P39462) (7); GsAHD, Adh from *Geobacillus stearothermophilus* (GenBank ID: P42328) (8); BsBSH-FDH, bacilithiol-depended Fdh from *Bacillus subtilis* (GenBank ID: NP_390579) (9); CbADH, Adh from *Clostridium beijerinckii* (GenBank ID: P25984) (10); TbADH, Adh from *Thermoanaerobacter brockii* (GenBank ID: P14941) (10); CgMSH-FDH, mycothiol-dependent Fdh from *Corynebacterium glutamicum* (GenBank ID: CAF18890) (11); RsGSH-FDH, glutathione- dependent Fdh from *Cereibacter sphaeroides* (GenBank ID: AAB09774) (12).

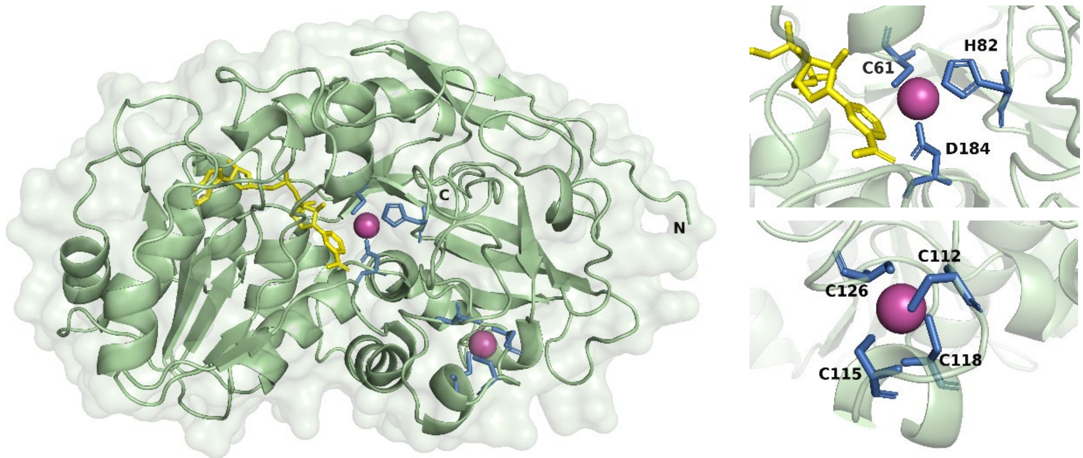

Figure S2: AlphaFold predicted structure of YycR. The enzyme is colored in green, with the NAD$^+$ cofactor in yellow and the catalytic and structural Zn^{2+} metal ion cofactors in pink. The catalytic (upper right) and structural (lower right) Zn^{2+} coordinating residues are shown in blue. The catalytic metal ion is deeply embedded in the structure. With the exception of the beginning of the N-terminal, all amino acids had a high prediction confidence (pLDDT > 90). The predicted template modeling (pTM) score and the interface predicted template modeling (ipTM) were 0.95 and 0.97, respectively. These values represent the accuracy of the entire structure, and values >0.8 represent confident high-quality prediction.

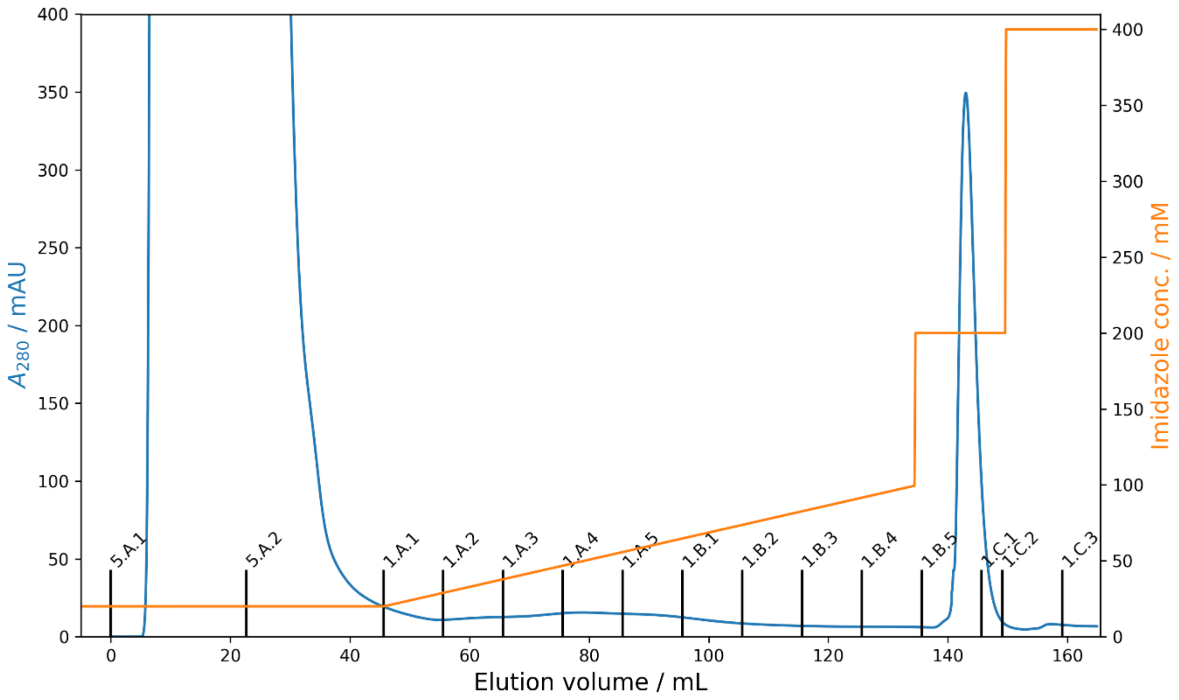

Figure S3: Chromatogram from the purification of YycR carried out using a 5 mL HisTrap HP column. The blue curve shows absorbance at 280 nm (A280) and the orange curve shows the gradient concentration of imidazole used during elution. Flow through (unbound protein) is found between 0–135 mL, whereas elution of YycR occurs at 130–150 mL and 100 mM imidazole. For illustration purposes, the flow-through peak (reaching a maximum of 3000 mAU) is truncated. The fraction names are labeled. Fraction 1.B5 is referred to as F1 and 1.C1 as F2 in the main text.

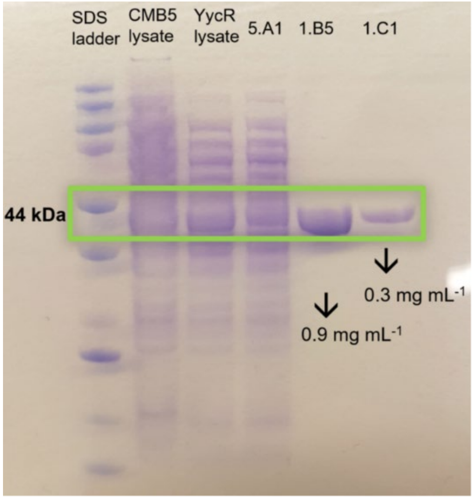

Figure S4: SDS-PAGE analysis of FPLC fractions resulting from YycR purification. CMB5 lysate represents the control *E. coli* T7 Express (pNIC-CH-CMB5), YycR lysate is the product of the *E. coli* T7 Express (pNIC-CH-*yycR*) strain, 5.A1 is the flow through (referred to as FT), 1.B5 (referred to as F1) and 1.C1 (referred to as F2) represent the fractions containing purified YycR. The green box marks bands corresponding to the molecular weight of YycR and the arrows direct to calculated protein concentrations of the associated fractions.

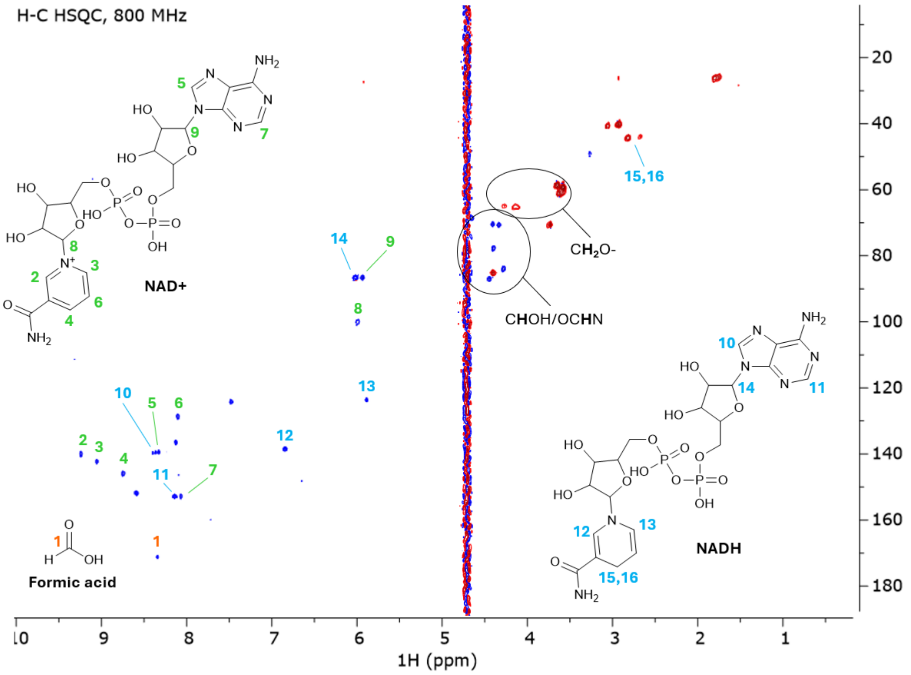

#	1H (ppm)	13C (ppm)
**1**	8.35	171.06
**2**	9.24	139.93
**3**	9.05	142.28
**4**	8.74	145.80
**5**	8.33	139.54
**6**	8.10	128.58
**7**	8.07	152.85
**8**	5.99	100.00
**9**	5.94	86.69
**10**	8.38	139.54
**11**	8.15	152.85
**12**	6.86	138.56
**13**	5.90	123.69
**14**	6.03	86.89
**15**	2.82	44.21
**16**	2.67	43.82

Figure S5: ^{1}H-^{13}C-HSQC spectrum (90% H_2O, 10% D_2O, 800 MHz) and chemical shift table of YycR reaction mixture. The original sample was prepared with 500 mM YycR, 10 mM NAD^+, 25 mM NaCl and 30 mM formaldehyde in 50 mM BISTRIS propane, pH 9.0, and the spectrum shows formation of NADH and formic acid. The NAD^+, NADH and formic acid cross-peaks are partially assigned based on (13).

Table S1: Screening YycR purification FPLC fractions based on the enzymatic activity of YycR. The fractions F1 and F2 contain purified YycR, FT stands for flow through, YycR crude extract is cell extract of *E. coli* T7 Express (pNIC-CH-*yycR*) strain, and CMB5 crude extract represents the crude extract of *E. coli* T7 Express (pNIC-CH-CMB5) control strain. Enzymatic assays were started by adding 1 mM formaldehyde to the reaction mixture and the enzymatic activity is reported in U mg^{-1}. A "n.d." stands for no enzymatic activity.

**FPLC fraction**	**YycR activity (U mg^{-1})**
YycR crude extract	0.022
CMB5 crude extract	n.d.
FT	n.d.
F1	0.046
F2	0.005

Table S2: The effect of various substrates on enzymatic activity of YycR. Enzymatic activity assays were performed at 37 °C using purified YycR (240 nM), 50 mM BISTRIS propane, 25 mM NaCl, pH 9. The enzymatic reactions were started by adding 1 mM of the substrate to the assay mix. The enzyme activities are reported in U mg^{-1}, "n.d." indicates no detected enzymatic activity.

**Substrate**	**YycR activity (U mg^{-1})**
Formaldehyde	0.70±0.05
Formic acid	n.d
Acetaldehyde	0.09± 0.02.
Glyoxal	n.d
Methanol	n.d.
Ethanol	n.d.
1-butanol	0.02±0.00

REFERENCES

1. Eddy SR. 2004. Where did the BLOSUM62 alignment score matrix come from? Nature Biotechnology 22:1035-1036.
2. Waterhouse AM, Procter JB, Martin DMA, Clamp M, Barton GJ. 2009. Jalview Version 2—a multiple sequence alignment editor and analysis workbench. Bioinformatics 25:1189-1191.
3. Marx CJ, Miller JA, Chistoserdova L, Lidstrom ME. 2004. Multiple formaldehyde oxidation/detoxification pathways in *Burkholderia fungorum* LB400. J Bacteriol 186:2173-8.
4. Ito K, Takahashi M, Yoshimoto T, D. T. 1994. Cloning and high-level expression of the glutathione-independent formaldehyde dehydrogenase gene from *Pseudomonas putida*. Journal of Bacteriology 176:2483-2491.
5. Zhang W, Chen S, Liao Y, Wang D, Ding J, Wang Y, Ran X, Lu D, Zhu H. 2013. Expression, purification, and characterization of formaldehyde dehydrogenase from *Pseudomonas aeruginosa*. Protein Expression and Purification 92:208-213.
6. Tang B, Xie F, Zhao W, Wang J, Dai S, Zheng H, Ding X, Cen X, Liu H, Yu Y, Zhou H, Zhou Y, Zhang L, Goodfellow M, Zhao GP. 2016. A systematic study of the whole genome sequence of *Amycolatopsis methanolica* strain 239^T provides an insight into its physiological and taxonomic properties which correlate with its position in the genus. Synth Syst Biotechnol 1:169-186.
7. Esposito L, Sica F, Raia CA, Giordano A, Rossi M, Mazzarella L, Zagari A. 2002. Crystal structure of the alcohol dehydrogenase from the hyperthermophilic Archaeon *Sulfolobus solfataricus* at 1.85Å resolution. Journal of Molecular Biology 318:463-477.
8. Ceccarelli C, Liang Z-X, Strickler M, Prehna G, Goldstein BM, Klinman JP, Bahnson BJ. 2004. Crystal structure and amide H/D exchange of binary complexes of alcohol dehydrogenase from *Bacillus stearothermophilus*: Insight into thermostability and cofactor binding. Biochemistry 43:5266-5277.
9. Nguyen TT, Eiamphungporn W, Mader U, Liebeke M, Lalk M, Hecker M, Helmann JD, Antelmann H. 2009. Genome-wide responses to carbonyl electrophiles in Bacillus subtilis: control of the thiol-dependent formaldehyde dehydrogenase AdhA and cysteine proteinase YraA by the MerR-family regulator YraB (AdhR). Mol Microbiol 71:876-94.
10. Korkhin Y, Kalb AJ, Peretz M, Bogin O, Burstein Y, Frolow F. 1998. NADP-dependent bacterial alcohol dehydrogenases: crystal structure, cofactor-binding and cofactor specificity of the ADHs of *Clostridium beijerinck*ii and *Thermoanaerobacter brockii*. Journal of Molecular Biology 278:967-981.
11. Lessmeier L, Hoefener M, Wendisch VF. 2013. Formaldehyde degradation in *Corynebacterium glutamicum* involves acetaldehyde dehydrogenase and mycothiol-dependent formaldehyde dehydrogenase. Microbiology (Reading) 159:2651-2662.
12. Barber RD, Rott MA, Donohue TJ. 1996. Characterization of a glutathione-dependent formaldehyde dehydrogenase from *Rhodobacter sphaeroides*. Journal of Bacteriology 178:1386-1393.
13. Wishart DS, Knox C, Guo AC, Eisner R, Young N, Gautam B, Hau DD, Psychogios N, Dong E, Bouatra S, Mandal R, Sinelnikov I, Xia J, Jia L, Cruz JA, Lim E, Sobsey CA, Shrivastava S, Huang P, Liu P, Fang L, Peng J, Fradette R, Cheng D, Tzur D, Clements M, Lewis A, De Souza A, Zuniga A, Dawe M, Xiong Y, Clive D, Greiner R, Nazyrova A, Shaykhutdinov R, Li L, Vogel HJ, Forsythe I. 2009. HMDB: a knowledgebase for the human metabolome. Nucleic Acids Res.37(Database issue):D603-10.
